# Arthritis and the Heart

**Published:** 1969-10

**Authors:** J. A. Cosh


### ARTHRITIS AND THE HEART

**J. A. Cosh**

The relationship between rheumatic fever and rheumatic heart disease has been recognised for nearly two centuries. In Bristol we are interested to know that one of the earliest observers in this field was Edward Jenner. He spoke to the Gloucestershire Medical Society on this subject in 1789 at one of their meetings at the Fleece Inn, Rodborough, but his observations were never published. Rheumatic heart disease has continued to be of importance in Bristol, both as a cause of morbidity, and as a subject for research and clinical study by the late Dr. Carey Coombs and by his successor Professor Bruce Perry. The recognition of heart lesions associated with other forms of rheumatic disease has followed only relatively recently, and some clarification of this rather confused subject will be attempted in this review.

Confusion has arisen between the lesions of rheumatic heart disease and those specifically due to rheumatoid disease, and others specifically associated with ankylosing spondylitis. To make matters worse, ankylosing spondylitis was for many years referred to in the American literature as " rheumatoid spondylitis ", although it is now everywhere accepted as being quite separate from rheumatoid arthritis. Lastly there is also the possibility that common conditions such as rheumatoid arthritis and rheumatic heart disease may co-exist by chance.

The conditions in which heart disease and chronic joint disease may be associated can be summarized as follows:—

1. Polyarthritis and coincidental heart disease of unrelated pathology,
2. Rheumatic heart disease and joint deformities resulting from repeated attacks of rheumatic fever (" Type Jaccoud "),
3. Systemic lupus erythematosus,
4. a) Ankylosing spondylitis with aortitis, aortic insufficiency and conduction defects,
   b) Similar lesions associated with Reiter's syndrome and psoriatic arthropathy,
5. Rheumatoid heart disease.

### COINCIDENCE

Chronic forms of polyarthritis, especially rheumatoid arthritis, are common, so that coincidental heart disease of different pathology must sometimes be expected. Confusion has sometimes arisen when valve lesions are found clinically, or at autopsy, in patients with rheumatoid arthritis. Often such valve lesions have been attributed to rheumatic heart disease, and the suggestion has been made that one condition predisposes to the other, or that the two pathological processes are related. This is no longer tenable, now that specific heart lesions are accepted as a part of rheumatoid disease, and of systemic lupus, and of spondylitis. In his review Bywaters (1950) concluded that rheumatic fever and rheumatoid arthritis were separate diseases, although they might occur by chance in the same subject. Similarly, there is no evidence that rheumatoid arthritis predisposes to the development of ischaemic or hypertensive heart disease, although these too may occur by chance in rheumatoid subjects.

### JACCOUD'S " ARTHRITIS "

Jaccoud in 1869 described a patient who, after 6 attacks of rheumatic fever, was left with heart lesions and with joint deformities in the hands. These were, essentially, ulnar deviation, and flexion deformities at the metacarpophalangeal joints, suggestive of rheumatoid arthritis. Although examples are rare, subsequent accounts (Bywaters, 1950; Thomas, 1955) make it clear that these joint deformities only superficially resemble rheumatoid, and that there is no radiological, serological, or histological evidence of rheumatoid arthritis.

### SYSTEMIC LUPUS ERYTHEMATOSUS (SLE)

Both polyarthritis and carditis are common manifestations of SLE. Arthritis and arthralgia occur in over 90% of patients: in a quarter of Dubois' (1966) series of 520 cases there was a polyarthritis resembling rheumatoid arthritis, and in others a migratory arthritis suggestive of rheumatic fever. The commonest cardiac lesion is pericarditis, diagnosed clinically in 30% of Dubois' series, but found in the majority of autopsied cases by Brigden *et al.* (1960). Valve lesions, mainly of mitral or aortic valves, may be recognised clinically, and are present in about half of autopsied cases. They take the form of small verrucous vegetations on both surfaces of affected valves, with endothelial thickening often spreading on to chordae or into the angle between cusp and mural endothelium. Macroscopically and microscopically they differ from the lesions of rheumatic heart disease, and do not often cause serious valve deformity; frank mitral or aortic stenosis is rare. The myocardium may also be involved, mainly by way of fibrinoid change in vessel walls in the connective tissue septa. Thus, SLE may mimic both rheumatoid arthritis and rheumatic fever, but its cardiac lesions, which are common, are histologically different both from the specific lesions of rheumatoid heart disease and from rheumatic heart disease.

### ANKYLOSING SPONDYLITIS

Characteristic aortic valve lesions have been recognised as an occasional visceral manifestation of ankylosing spondylitis since the original observation of Bauer *et al.* (1951).

Ansell *et al.* (1958) could find only two examples however in a series of 222 patients. Graham and Smythe (1958) reported 21 cases of non-syphilitic " lone aortic insufficiency " among 519 Canadian veterans with spondylitis, and noted that the incidence was higher in those with a longer duration of spondylitis. In men with a 5 year duration of disease, aortic insufficiency was found in 1%, whereas among those with a 30 year history the incidence was 10%. In those who had peripheral joint involvement as well as spondylitis, the incidence was higher still, i.e. aortic valve lesions are mainly associated with severe and widespread spondylitis of long duration. A further review of the subject was reported, from Bristol, by Crow (1960). Aortic valve insufficiency is due to fibrous thickening and scarring of the valve cusps which do not become greatly adherent or stenotic. A contributory feature may be dilatation of the aortic valve ring resulting from a low grade aortitis of the ascending aorta with damage to elastic and muscle fibres in the media. Fibrosis may extend downwards into the heart to involve the conducting tissue and produce heart block (Julkunen, 1964). Transient pericarditis has also been described in the early stages of ankylosing spondylitis.

**Case 1. Aortic insufficiency with ankylosing spondylitis following Reiter's syndrome.**

A soldier aged 37 developed Reiter's syndrome after dysentery, and 12 years later was found to have ankylosing spondylitis; the knees were involved as well as the spine. Four years later aortic insufficiency was discovered. After 2 more years he had a number of attacks of left ventricular failure, and died in congestive failure at the age of 55.

**Autopsy** confirmed the presence of ankylosing spondylitis in the spine, sternoclavicular joints, and knees. The left ventricle was hypertrophied and dilated. The aortic valve cusps were fused at their commissures, thickened at their free margins, and covered with a deposit of fibrous tissue extending from the base of the cusps into the aortic sinuses. In the aorta there was chronic inflammation with patchy destruction of elastic fibres, infiltration with lymphocytes, and endarteritis obliterans.

### REITER'S SYNDROME AND PSORIATIC ARTHROPATHY

A number of observers have described pericarditis and temporary conduction defects in the acute stage of Reiter's syndrome. Attention was first drawn to the subsequent development of aortic insufficiency by Csonka (1961) and Rodnan *et al.* (1964). They found aortic valve lesions resembling those seen in spondylitic heart disease.

Ankylosing spondylitis can be regarded as a pattern of joint disease, of unknown cause, sometimes following disease of the colon (ulcerative colitis or Crohn's disease) and sometimes following Reiter's syndrome (which itself may begin with either urethritis or dysentery). In such examples as Case 1 here, and case 27 of Dixon (1960), aortic insufficiency was associated with spondylitis which followed Reiter's syndrome. However, spondylitis is not necessarily part of the association.

Psoriatic arthropathy is now accepted as a form of chronic polyarthritis differing in a number of respects from classical rheumatoid arthritis. It has no specific association with heart lesions, but Wright and Reed (1964) pointed out resemblances between psoriatic arthropathy and Reiter's syndrome, and in two of the 11 patients they described there were valve lesions. One had aortic insufficiency and the other mitral and aortic stenosis.

### RHEUMATOID HEART DISEASE

Since the early descriptions of Charcot (1881) and Still (1896) pericarditis has been associated with rheumatoid arthritis, both juvenile and adult. The concept of specific granulomatous heart lesions in rheumatoid was put forward by Baggenstoss and Rosenberg in 1944. Other descriptions followed, and a good review of the pathology of these and other lesions associated with rheumatoid was given by Cruickshank (1958). Such accounts of visceral damage accompanying rheumatoid arthritis make it appropriate to speak of

" rheumatoid disease ", emphasizing that this is a disorder that can do very much more than damage the joints (Sinclair and Cruickshank, 1956).

Types of heart lesion in rheumatoid disease may be summarized as follows:

(a) Granuloma, resembling the subcutaneous nodule of rheumatoid arthritis
— endocardial, affecting mitral or aortic valves
— myocardial, affecting conducting tissue
— epicardial

(b) Pericarditis, of non-specific histology

(c) Endocarditis of non-specific histology

(d) Myocarditis of non-specific histology

(e) Coronary arteritis

As the last three are basically histological diagnoses of little clinical significance, further discussion here will be confined to (a) and (b).

*Granuloma*

The incidence of rheumatoid granulomata is probably 5%, although there is considerable variation in the figures given in reported autopsy studies. They are only found in patients who have a long history of chronic nodular rheumatoid arthritis, in whom the Rose-Waaler test is usually strongly positive. The histological structure of the granuloma resembles that of the classic rheumatoid nodule: there is a central area of fibrinoid necrosis, surrounded by a ring of palisaded fibroblasts, beyond which is a round cell reaction of lymphocytes and plasma cells.

In the case of endocardial lesions, which are usually in or near the aortic and mitral valve rings, the appearance of the granuloma is modified by its position. The functional result is aortic insufficiency of a progressive nature, or mitral stenosis, which is not likely to become very severe. During life it is impossible to do more than suspect that such lesions are an integral part of rheumatoid disease, rather than due to coincident rheumatic heart disease.

**Case 2. Rheumatoid aortic and mitral valve lesions.**

A man of 43 developed seropositive rheumatoid arthritis, and after ten years was admitted to the Royal National Hospital for Rheumatic Diseases, Bath, for physio- and hydrotherapy. He was noted to have the murmurs of aortic insufficiency and of mitral stenosis with insufficiency; these were thought to be due to coincidental rheumatic heart disease. Four years later he was admitted as an emergency to the Bristol Royal Infirmary in left ventricular and congestive heart failure due to severe aortic insufficiency, from which he died. At autopsy the left ventricle was greatly hypertrophied and dilated with an endocardial jet lesion due to aortic valve regurgitation below one of the cusps; this cusp had a nodular thickening upon it with the histological features of a rheumatoid granuloma. There was central necrosis, with surrounding fibrosis and round cell infiltration and some secondary calcification. A similar nodular lesion was found on one cusp of the mitral valve, and there was a slight degree of mitral valve stenosis.

When granulomata are situated in the myocardium they are likely to be without clinical effect unless they lie in the region of the conducting tissue or are sufficiently numerous to constitute a form of low grade myocarditis. The development of heart block has been described in patients with chronic rheumatoid disease, and the case report which follows illustrates this as well as low grade myocarditis.

**Case 3. Rheumatoid granuloma in myocardium; first degree heart block.**

A woman of 62 developed seropositive rheumatoid arthritis which was progressive and disabling; after three years, corticosteroid therapy was started. After four more years she began to have paroxysmal nocturnal dyspnoea; gallop rhythm was heard, and the ECG showed first-degree heart block (PR 0.28 sec., compared with 0.16 sec. two years earlier). She died with bronchopneumonia. At autopsy a number of rheumatoid granulomata up to 5 mm. in diameter were found in the interventricular septum and one lay on the epicardial surface of the LV. One of those in the septum lay immediately below the AV node, which was invaded by the round cell reaction around the granuloma. Elsewhere throughout the myocardium was a patchy diffuse round cell infiltration of lymphocytes and plasma cells (also see Kirk and Cosh, 1969, case 10).

*Pericarditis*

Many reports of pericarditis in rheumatoid disease have appeared in the past decade. Attention has been focused upon it as a possible cause of constrictive pericarditis, which may require surgical relief (Kennedy *et al.*, 1966). However, this is unusual, for rheumatoid pericarditis is generally unobtrusive and often symptomless, and may only be detected incidentally during the course of clinical examination (Kirk and Cosh, 1969).

In reports of autopsy studies of rheumatoid arthritis the incidence of pericarditis is roughly 30%. Pericardial adhesions are often incomplete and not very dense, or there may be a lightly adherent fibrinous pericarditis (' bread and butter '). As a clinical finding, however, pericarditis is not often noted. This discrepancy prompted Julian Kirk and myself to examine repeatedly and closely 100 consecutively admitted patients with chronic rheumatoid arthritis at the Royal National Hospital for Rheumatic Diseases. To our surprise, we found ten patients with pericarditis, implying that pericarditis is commoner than is believed. With 21 other clinical examples, and two more in which pericarditis was an unexpected finding at autopsy, our final series totalled 33.

Pericarditis usually occurs in seropositive cases of rheumatoid arthritis, and can be found at any stage. It can even, like pleurisy, appear as a presenting manifestation, and often pericarditis and pleurisy occur together. It can persist in mild degree for weeks or months. The commonest symptoms in our series were pain, either pleural or pericardial in type, and dyspnoea, although in 15 of our 33 it gave rise to no symptoms. The commonest physical sign was a pericardial friction rub, present in 25, while in others the diagnosis was based on serial electrocardiographic changes, or changes in the size of the heart shadow on X-ray. Only four had the classical ECG pattern of pericarditis, and 12 had no ECG changes at all. This indicates that the inflammatory reaction is not intense and does not penetrate far into the myocardium. Eighteen patients had a low fever while pericarditis was present. Twelve had simultaneous pleurisy or pleural effusion. There was evidence of a pericardial effusion in six, which produced tamponade in three. Aspiration of fluid was carried out in only one, with relief of the signs of tamponade. In this fluid there was a low glucose content (10 mg/100 ml.); this had been noted too in rheumatoid pleural effusions. Only one patient proceeded to constrictive pericarditis:—

**Case 4. Pleural and pericardial effusions: constrictive pericarditis.**

A woman of 53 developed classical seropositive rheumatoid arthritis, which was progressive, and for which she was given corticosteroids in the first year of the disease. Five years later she had a haematemesis from a gastic ulcer. While she was in hospital recovering from this, pericardial friction was heard. A large pericardial effusion and a right pleural effusion developed, and the latter was aspirated (750 ml). She later developed a smaller left pleural effusion. She improved and left hospital but two months later had leg oedema and hepatomegaly. Within seven months of the discovery of pericarditis she had obvious constrictive pericarditis, and was referred for surgery. A greatly thickened pericardium was excised, with relief, but she unfortunately died on the first post-operative day. (Also described by Harrold, 1968, case 1).

*Treatment of pericarditis*

In our experience, steroids did not necessarily influence the duration of pericarditis. Aspiration is indicated for a large effusion, particularly if it is causing cardiac tamponade, and instillation of hydrocortisone into the pericardium at the time of aspiration is probably helpful, though we have no proof of this. The course of events in Case 4 suggests that aspiration of the effusion might have halted the development of constrictive pericarditis, in the same way that aspiration of a pleural effusion may lessen the degree of subsequent pleural thickening.

My thanks are due to my colleagues at the Royal National Hospital for Rheumatic Disease, Bath, for allowing me to study their patients, to Professor C. Bruce Perry, for permission to quote Case 1, to Dr. J. E. Cates, in respect of Case 2, and to Dr. O. C. Lloyd for the pathological reports on Cases 1 and 2.

### REFERENCES

Ansell, B. M., Bywaters, E. G. L., and Doniach, I. (1958). Brit. Heart J. *20*, 507.

Baggenstoss, A. H., and Rosenberg, E. F. (1944) Arch. Path. *37*, 54.

Bauer, W., Clark, W. S., and Kulka, J. P. (1951) Ann. rheum. Dis. *10*, 470.

Brigden, W., Bywaters, E. G. L., Lessof, M. H., and Ross, I. P. (1960) Brit. Heart J. *22*, 1.

Bywaters, E. G. L. (1950) Brit. Heart J. *12*, 101.

Crow, R. S. (1960) Brit. Med. J. *2*, 271.

Cruickshank, B. (1958) J. Path. Bact. *76*, 223.

Csonka, G. W., Litchfield, J. W., Oates, J. K., and Willcox, R. R. (1961) Brit. Med. J. *1*, 243.

Dixon, A. St. J. (1960) Ann. Rheum. Dis. *19*, 209.

Dubois, E. L. (1966) ' Lupus Erythematosus '. McGraw Hill, New York.

Graham, D. C. and Smythe, H. A. (1958) Bull. Rheum. Dis. *9*, 171.

Harrold, B. P. (1968) Brit. Med. J. *1*, 290.

Jaccoud, F. S. (1869) Leçons de Clinique Medicale a l'Hôp. de la Charité. 2nd Edition, Paris.

Julkunen, H. (1964) Acta. Med. Scand. *176*, 401.

Kennedy, W. P. U., Partridge, R. E. H., and Matthews, M. B. (1966) Brit. Heart J. *28*, 602.

Kirk, J. A., and Cosh, J. A. (1969) Quart. J. Med. (In press).

Rodnan, G. P., Benedek, T. G., Shaver, J. A., and Fennel, R. H. (1964). J. Amer. Med. Assn. *189*, 889.

Sinclair, R. J. G., and Cruickshank, B. (1956). Quart. J. Med. *25*, 313.

Thomas, A. E. (1955) Ann. Rheum. Dis. *14*, 259.

Wright, V. and Reed, W. B. (1964). Ann. Rheum. Dis. *23*, 12.

